# Comorbidity and osteoporotic fracture: approach through predictive modeling techniques using the OSTEOMED registry

**DOI:** 10.1007/s40520-022-02129-5

**Published:** 2022-04-18

**Authors:** María Begoña Coco Martín, Luis Leal Vega, José Antonio Blázquez Cabrera, Amalia Navarro, María Jesús Moro, Francisca Arranz García, María José Amérigo, Manuel Sosa Henríquez, María Ángeles Vázquez, María José Montoya, Manuel Díaz Curiel, José Manuel Olmos, José Luis Pérez Castrillón, OSTEOMED Group, José Filgueira Rubio, Pilar Sánchez Molini, José María Aguado Caballero, Dolors Armengol Sucarrats, María Luz Calero Bernal, Begoña de Escalante Yanguas, Nerea Hernández de Sosa, José Luis Hernández, Julia Jareño Chaumel, María José Miranda García, Mercedes Giner García, Cristina Miranda Díaz, Rafael Cotos Canca, Juan Carlos Cobeta García, Francisco Javier Rodero Hernández, Raimundo Tirado Miranda

**Affiliations:** 1 Group of Applied Clinical Neurosciences and Advanced Data Analysis, Department of Medicine, Dermatology and Toxicology, University of Valladolid, Valladolid, Spain; 2 Internal Medicine Service, Hospital General Universitario de Albacete, Albacete, Spain; 3 Internal Medicine Service, Hospital Universitario Infanta Leonor, Madrid, Spain; 4 Internal Medicine Service, Hospital Clínico San Carlos, Madrid, Spain; 5 Internal Medicine Service, Hospital Universitario Insular de Gran Canaria, Las Palmas de Gran Canaria, Spain; 6 Departament of Medicine, University of Seville, Seville, Spain; 7 Internal Medicine Service, Fundación Jiménez Díaz, Madrid, Spain; 8 Internal Medicine Service, Hospital Universitario Marqués de Valdecilla, Cantabria, Spain; 9 Internal Medicine Service, Hospital Universitario Río Hortega, Valladolid, Spain

**Keywords:** Osteoporosis, Osteoporotic fractures, Anti-osteoporotic treatment, Comorbidities, Logistic regression, Artificial neural network

## Abstract

**Purpose:**

To examine the response to anti-osteoporotic treatment, considered as incident fragility fractures after a minimum follow-up of 1 year, according to sex, age, and number of comorbidities of the patients.

**Methods:**

For this retrospective observational study, data from baseline and follow-up visits on the number of comorbidities, prescribed anti-osteoporotic treatment and vertebral, humerus or hip fractures in 993 patients from the OSTEOMED registry were analyzed using logistic regression and an artificial network model.

**Results:**

Logistic regression showed that the probability of reducing fractures for each anti-osteoporotic treatment considered was independent of sex, age, and the number of comorbidities, increasing significantly only in males taking vitamin D (OR = 7.918), patients without comorbidities taking vitamin D (OR = 4.197) and patients with ≥ 3 comorbidities taking calcium (OR = 9.412). Logistic regression correctly classified 96% of patients (Hosmer–Lemeshow = 0.492) compared with the artificial neural network model, which correctly classified 95% of patients (AUC = 0.6).

**Conclusion:**

In general, sex, age and the number of comorbidities did not influence the likelihood that a given anti-osteoporotic treatment improved the risk of incident fragility fractures after 1 year, but this appeared to increase when patients had been treated with risedronate, strontium or teriparatide. The two models used classified patients similarly, but predicted differently in terms of the probability of improvement, with logistic regression being the better fit.

## Introduction

Comorbidity is defined as one or more coexisting conditions in a patient with an index disease, and is a risk factor for frailty and disability [1, 2]. In this regard, geriatric patients, who are increasingly complex, are often multi-pathological, requiring healthcare professionals to take a holistic view of their care [3, 4]. Comorbidity may act as a confounding factor, altering the detection, prognosis, treatment, and outcome of the index disease in these patients.

Osteoporosis is a common disease in the elderly, with hip fracture being the most serious complication, with a high associated morbidity and mortality rate [5]. Fractures are a major health problem, whose incidence clearly increases with age [6]. Many risk factors determine the appearance of fractures, and they are broadly classified into those that with a deleterious effect on bone and those that increase the risk of falls [7]. These patients have a high prevalence of comorbidities that may condition the clinical evolution and therapeutic response to osteoporosis. It is estimated that 80% of osteoporotic patients have at least one chronic comorbidity. For this reason, some fracture risk scales, such as FRAX^®^ or QFracture^®^, include elements of comorbidity in their items [8, 9].

Therefore, it is important to characterize the evolution of osteoporosis, as measured by changes in bone mineral density (BMD) and the occurrence of fractures, according to the number of comorbidities that patients present [10].

This study used a cohort of osteoporotic patients followed over time and evaluated their response to the prescribed anti-osteoporotic treatment, determined by the occurrence of vertebral, humerus or hip fractures after a follow-up of ≥ 1 year, according to their sex, age, and number of comorbidities. In addition to conventional (logistic regression), we used an artificial neural network model to develop an algorithm that calculates the probability of improvement in fracture risk for each patient based on sex, age, number of comorbidities and anti-osteoporotic treatment received.

To our knowledge, this is the first time that two differing predictive modeling techniques have been used to examine the response to anti-osteoporotic treatment as a function of sex, age, and number of comorbidities. This may aid clinical decision-making and help healthcare professionals dealing with elderly osteoporotic patients with a large number of associated diseases.

## Materials and methods

### Study design

This retrospective observational study examined whether the response to anti-osteoporotic treatment, as determined by incident fragility fractures after a minimum follow-up of 1 year, was influenced by sex, age, and the number of comorbidities. We analyzed the number of comorbidities, the anti-osteoporotic treatment prescribed and the occurrence of vertebral, humerus or hip fractures at two visits, a baseline visit when patients were first referred to the internal medicine consultation for the evaluation or diagnosis of osteoporosis or fractures, and a follow-up visit at ≥ 1 year.

### Study population

The study population comprised 993 patients from the Osteoporosis in Internal Medicine (OSTEOMED) registry [912 females (91.84%) and 81 males (8.16%), mean age 65.39 ± 11.15 years] with matching baseline and follow-up data.

The OSTEOMED registry is composed of patients who attended internal medicine consultations in 23 Spanish hospitals for the evaluation and diagnosis of osteoporosis or the presence of fractures between 2012 and 2017 [11].

The patients included in this registry were referred from primary care, other hospital services and from other internal medicine consultations.

We included patients diagnosed with osteoporosis according to the densitometric criteria established by the World Health Organization (WHO) (T-score < -2.5 in any location) or typical fragility fractures (vertebral, humerus or hip) independently of the BMD [12, 13]. Patients with malignancies, a life expectancy of < 1 year who were aged > 90 years were excluded, as their follow-up in the proposed way was considered unfeasible.

The study patients were followed up according to standard clinical practice, meaning that no additional diagnostic tests or therapeutic interventions were performed. However, all patients received an information sheet on the objectives of the study and signed a written informed consent prior to the collection of clinical data.

### Study variables

The variables collected came from a medical history specifically focused on osteoporosis and fractures. Fractures, comorbidities and the prescribed anti-osteoporotic treatment were obtained from the patients’ medical records and entered in a specific electronic database by trained research staff from the participating centers.

Comorbidities recorded included hypertension, dyslipidemia, diabetes, hypothyroidism, hyperthyroidism, hyper-calciuria, nephrolithiasis, coronary heart disease, chronic kidney disease, chronic obstructive pulmonary disease, interstitial lung disease, coeliac disease, Crohn’s disease, breast cancer, prostate cancer, temporal arteritis, rheumatic polymyalgia and rheumatoid arthritis.

Two numerical variables were created for the total number of vertebral, humerus and hip fractures confirmed by radiography at baseline and follow-up visits, respectively.

We created a categorical variable named Fractures Variation, which took the value of 0 when the number of fractures between the baseline and follow-up visits decreased or stayed the same (improvement) and the value of 1 when the number of fractures increased (worsening).

Another categorical variable called Comorbidities was created, which took the value 0 when the patient had no comorbidities at baseline, value 1 when the patient had one comorbidity, value 2 when the patient had two comorbidities and value 3 when the patient had ≥ 3 comorbidities. Other categorical variables created were Sex (male or female), Age (< 65, 65 to 75 and > 75 years) and Treatments, which took the value 0 or 1 depending on whether the patient was prescribed calcium, vitamin D, alendronate, risedronate, strontium, teriparatide (PTH) or denosumab.

### Statistical analysis

The paired sample *t* test was used to test whether the number of vertebral, humerus and hip fractures between baseline and follow-up visits were equal and the Wilcoxon signed-rank test was used for this purpose when the relationship shown by the paired *t* test was unclear.

Contingency tables and logistic regression analyses were used to determine whether the likelihood of improving the risk of fracture depended on Sex, Age or the number of Comorbidities. As a measure of the goodness-of-fit of the model, the Hosmer and Lemeshow test was used to compare the estimated values with the observed values. Another measure of goodness-of-fit used was the percentage of cases correctly classified by the model, considering outliers.

Another key measure calculated was the odds ratio (OR) associated with each anti-osteoporotic treatment prescribed, which reflects how many times the probability of fracture risk improvement is greater than the probability of fracture risk worsening when received (values > 1 mean the probability of improvement increases, and values < 1 mean the probability of improvement decreases). The greater the OR exceeding 1, the greater the probability of fracture risk improvement with each prescribed treatment.

The model was complemented with an artificial neural network model to predict the probability of improving a patient’s fracture risk given a particular treatment [14–16]. The steps followed for the design of the neural network model were as follows: selection of the variables of interest (age, sex, number of comorbidities, incident fragility fractures and anti-osteoporotic treatment prescribed), data processing, creation of the groups, and selection and construction of the neural network model. To find the most effective model, we created different models by modifying the learning rate and the momentum factor. The cut-off point that was selected as a threshold to decide whether to classify patients as improving or not was 0.5. Of the models tested, the best fit was obtained for a multi-layer Perceptron network model with a downward gradient and cut-off point of 0.5, a learning rate of 0.5, a momentum of 1.0 and a relative minimum change in training error of 0.001. The function used in the hidden layer was a hyperbolic tangent function, while in the output layer, it was a SoftMax function.

To obtain the model, the sample of 993 patients was divided into two groups: the training group (which was used in the learning phase), and the test group (which was used in the trial phase to demonstrate the functioning of the network). To do this, the training group consisted of 70% of the patients so that the network could iteratively adjust the weights, and the validation group included 30% of the patients.

## Results

### Population

The Sex and Age of the 993 patients included in the analysis are shown in Table 1.

**Table 1:** Sex and age of patients

Patients	993
Sex	
Females	912
Males	81
Age	
< 65 65–75	492 292
> 75	209

The number of Comorbidities presented by patients according to Sex and Age is shown in Table 2.

**Table 2:** Number of comorbidities presented by patients

Co-morbidities	0	1	2	≥ 3
Females	349	327	175	61
Males	36	25	13	7
Total	385	352	188	68
< 65	228	170	71	23
65–75	87	105	69	31
> 75	70	77	48	14
Total	385	352	188	68

### Fractures

The number of vertebral, humerus and hip fractures patients had at baseline and follow-up visits is shown in Table 3.

**Table 3:** Number of fractures presented by patients at baseline and follow-up visits

Fractures	Baseline (*n*)	Follow-up (*n*)
Vertebral	41	42
Humerus	13	5
Hip	165	8

### Treatments

The anti-osteoporotic treatments patients received between baseline and follow-up visits are shown in Table 4.

**Table 4:** Treatments prescribed to patients at the baseline visit

Treatments	Patients (*n*)	Patients (%)
Calcium	724	72.9
Vitamin D	808	81.4
Alendronate	103	10.4
Risedronate	139	14
Strontium	48	4.8
PTH	120	12.1
Denosumab	179	18

Of the 993 patients in the cohort, 502 (50.55%) were receiving anti-osteoporotic treatment prior to the baseline visit: 400 calcium (40.28%), 428 vitamin D (43.1%), 146 alendronate (14.7%), 93 risedronate (9.36%), 53 strontium (5.33%) and 50 PTH (5.03%).

Using analysis of variance (ANOVA), we studied whether the mean number of fractures at the baseline visit differed significantly according to the number of Comorbidities, and found that it not (*p* value = 0.258, IC 95%).

The paired sample *t* test used to test whether the mean number of fractures at the follow-up visit increased from the baseline visit showed a difference in means (*p* value < 0.001), confirming that the number of fractures recorded at the follow-up visit was significantly lower than the number of fractures recorded at baseline. The non-parametric Wilcoxon signed-rank test for equality of means in paired samples confirmed the difference in means (*p* value < 0.001), and therefore the number of fractures decreased significantly between the baseline and follow-up visits.

A logistic regression model was used to explain the probability of improving the risk of fracture as a function of the variable Treatments, in addition to the variables Sex, Age and number of Comorbidities. However, before including these in the model, we studied their relationship with the variable FracturesVariation using contingency tables, and found the probability of improving the risk of fracture was independent of Sex, Age and the number of Comorbidities.

The resulting logistic regression model correctly classified 96% of patients, classified all cases as improving and the Hosmer and Lemeshow test was 0.492, thus accepting the hypothesis that the model obtained explains the data observed. Therefore, Sex, Age and number of Comorbidities did not, in general, influence the likelihood that a given anti-osteoporotic treatment improved the risk of vertebral, humerus or hip fracture after a follow-up period of ≥ 1 year, although this appeared to be lower when patients have been treated with risedronate, strontium and PTH.

According to the model obtained, the probability of improving the risk of fracture was equal to:

1/(1 + e^–(2,814+0,170*Ca+0,137*VitD−0,637*Alen+0,724*Rise+0,729*Stron+0,609* PTH−0,10*Denos)^)

The results of the contingency table analysis and logistic regression model used to determine which Treatments increased the likelihood of improving fracture risk according to Sex are summarized in Table 5.

**Table 5:** Evolution of fractures according to Sex and Treatment

	Women	Men
Patients	913	81
Fracture risk improvement	874	77
% Fracture risk improvement	96%	95%
Fracture risk improvement was independent of Sex (contingency tables)		
Goodness-of-fit of the model	*p* = 0.669	*p* = 0.979
% Correctly classified cases	96%	95%
Outliers	39 (*p* > 0.95)	3 (*p* > 0.85)
Calcium (Odds ratio)	1.217	0.581
Vitamin D (Odds ratio)	0.999	7.918
Alendronate (Odds ratio)	0.495	2.007
Risedronate (Odds ratio)	2.343	3.376
Strontium (Odds ratio)	1.997	N/A
PTH (Odds ratio)	1.423	N/A
Denosumab (Odds ratio)	0.934	2.442

N/A: It was not possible to calculate the OR due to the lack of cases

The results of the contingency table analysis and logistic regression model used to determine which Treatments increased the likelihood of improving fracture risk according to Age are summarized in Table 6.

**Table 6:** Evolution of fractures according to Age and Treatment

	< 65	65–75	> 75
Patients	492	292	209
Fracture risk improvement	472	281	197
% Fracture risk improvement	96%	96%	94%
Fracture risk improvement was independent of Age (contingency tables)			
Goodness-of-fit of the model	*p* = 0.422	*p* = 0.983	*p* = 0.703
% Correctly classified cases	96%	96%	94%
Outliers	20 (*p* > 0.9)	11 (*p* > 0.8)	12 (*p* > 0.8)
Calcium (Odds ratio)	0.997	1.497	1.633
Vitamin D (Odds ratio)	0.856	1.278	1.604
Alendronate (Odds ratio)	2.459	0.265	0.113
Risedronate (Odds ratio)	N/A	0.528	0.439
Strontium (Odds ratio)	N/A	N/A	0.163
PTH (Odds ratio)	1.316	N/A	1.134
Denosumab (Odds ratio)	2.478	0.727	0.266

N/A: It was not possible to calculate the OR due to the lack of cases

The results of the contingency table analysis and logistic regression model used to determine which Treatments increased the likelihood of improving fracture risk according to the number of Comorbidities are summarized in Table 7.

**Table 7:** Evolution of fractures according to Comorbidities and Treatment

	0	1	2	≥ 3
Patients	385	352	188	68
Fracture risk improvement	367	341	180	64
% Fracture risk improvement	95%	97%	96%	94%
Fracture risk improvement was independent of Comorbidities (contingency tables)				
Goodness-of-fit of the model	*p* = 0.782	*p* = 0.978	*p* = 1.000	*p* = 1.000
% Correctly classified cases	95%	97%	96%	94%
Outliers	16 (*p* > 0.8)	12 (*p* > 0.9)	8 (*p* > 0.8)	3 (*p* > 0.8)
Calcium (Odds ratio)	0.368	2.441	1.646	9.412
Vitamin D (Odds ratio)	4.197	0.298	0.000	2.124
Alendronate (Odds ratio)	0.585	0.292	N/A	0.000
Risedronate (Odds ratio)	N/A	1.789	N/A	0.000
Strontium (Odds ratio)	N/A	N/A	0.980	0.202
PTH (Odds ratio)	1.350	1.567	N/A	0.202
Denosumab (Odds ratio)	0.889	0.991	2.465	0.000

N/A: It was not possible to calculate the OR due to the lack of cases

The artificial neural network model correctly classified 95% of patients, classified all patients as improving and had an area under the ROC curve of 0.6, showing the model’s fit with reality. Comparison of the probability of improvement of each patient according to the logistic regression model and the artificial neural network model showed that both models correctly classified ≈ 95% of patients (96% in the logistic regression), both classified all patients as improving, but there was no positive correlation and there was a difference in means between the two variables (paired sample *t* test). Therefore, we can conclude that both models classified similarly but predicted differently in terms of the probability of improvement, with the logistic regression model being the better fit.

## Discussion

We analyzed the effect of anti-osteoporotic treatments in reducing fractures after a minimum follow-up period of 1 year according to sex, age and number of comorbidities. We used two predictive techniques focused on modeling (logistic regression and artificial neural network). Logistic regression has a more rigid model structure and a set of assumptions and hypotheses that must be met prior to analysis, which is not the case with artificial neural networks. Nevertheless, when in addition to looking for the underlying model that explains the relationships between the dependent and independent variables, the interpretation of the model is important, logistic regression must be used.

The logistic regression model was the best fit and the most informative, as it gave us the probability of improvement for each patient and also how many times the probability of improvement was greater than that of worsening when receiving a given treatment (OR), so we can determine which treatments help to improve the risk of fracture the most and which do not. The artificial neural network model also showed a good measure of goodness-of-fit, although it only provided the probability of improvement for each patient.

Comorbidity was assessed in terms of the number of diseases associated with the index disease, osteoporosis [17]. This methodology was used by Silverman et al. [18] to analyzed the influence of comorbidities on fractures in the FREEDOM trial. This trial found no relationship between this and the prevalent fractures observed during the study, but did find a relationship with previous fractures. We found no relationship with either previous or prevalent fractures, nor did we find an association with the FRAX^®^ index, a scale predictive of fracture risk.

Another study evaluated the usefulness of comorbidity indices (including the number of associated diseases) to predict different events in various chronic diseases, including osteoporosis, but none proved useful for this purpose [19]. One index evaluated was the Charlson comorbidity index (CCI), one of the most commonly used in this type of study, although its performance is better when applied to a database with ICD-9 coded diagnoses [20].

Given these studies, measuring comorbidity by the number of associated diseases validated. Most comorbidity indices are not designed to assess cohorts, so a pragmatic index such as that used in our study may be useful. The aim of these in chronic pathologies is to control confounding factors between health outcomes and the index disease, such as fractures in the case of osteoporosis.

Most studies associate comorbidity with an increased risk of prevalent fractures, which are those that patients present at the time of care. However, not all studies are conclusive. In the GLOW study, a cohort including more than 50,000 women over the aged ≥ 55 years, a positive correlation was found between fracture risk and comorbidities, although these included rheumatoid arthritis and neurological pathologies, such as Parkinson’s disease, multiple sclerosis, and stroke [21]. In 5500 women from the same cohort selected for a 3 years follow-up, no increased risk of fracture was associated with these comorbidities [22]. However, studies have shown such an association, such as the Women’s Health Initiative (WHI) randomized controlled trial, in which an OR > 2 was observed in females with ≥ 3 comorbidities after 7 years of follow-up [23]. We found no association between the number of comorbidities and prevalent fractures. This may be due to the age of the sample, a young population in the context of osteoporosis, and the small number of patients with ≥ 3 comorbidities.

The other aspect evaluated was the efficacy of the prescribed treatment according to comorbidities. Few studies have evaluated the response to treatment considering comorbidity as a whole. Most assessed the influence of single diseases or specific groups of diseases, such as neurological or cardiovascular diseases. Considered in isolation, Parkinson’s disease was associated with a worse response to treatment [21], while, taken together, a study of the effect of cardiovascular comorbidities in osteoporotic women treated with risedronate found no significant changes in the response to treatment based on the presence or absence of these comorbidities [24].

Analysis of the response to treatment showed that the best response was obtained in males, in patients without comorbidities taking vitamin D and in patients with three or more comorbidities taking calcium. This is because these are patients at low risk of fracture and many of them might have been on therapeutic holidays having previously received bisphosphonates, which bind to the bone and are released with the activation of bone remodeling, re-exerting their effect [25], so this would constitute a limitation with respect to the study analysis. The criteria for indicating therapeutic holidays are an absence of fractures and a BMD in the non-osteoporotic range [26]. Administration of vitamin D helps to maintain blood levels above 10 ng/ml, which has been associated with a lower risk of fractures and osteomalacia [27], although other authors have placed the vitamin D threshold for ensuring the benefit of bisphosphonates in reducing fractures at higher levels [28, 29].

Another key aspect to consider is adherence to treatment, as polypharmacy in patients with many comorbidities who do not perceive an immediate benefit from anti-osteoporotic treatment may lead to increased drug discontinuation, as observed in previous studies [7, 30].

The main limitations of the study are the relatively short duration of follow-up (≥ 1 year), the low age and degree of comorbidity of the patients and the fact that slightly more than half (50.55%) were taking anti-osteoporotic treatment prior to baseline (including bisphosphonates, which may have a residual effect due to their long biological half-life). The strengths of the study included the sample size and the robustness of the statistical methodology employed, since the same results were obtained by analyzing the data with two different statistical software packages (SPSS 27.0 and R 4.1.2).

In conclusion, sex, age and the number of comorbidities are not associated with a worse response to prescribed anti-osteoporotic treatment when determined by incident fragility fractures (vertebral, humerus and hip) after a follow-up period of ≥ 1 year. The two models used classify patients similarly (improvement or non-improvement), but predict differently in terms of the probability of improvement, with the logistic regression model being the better fit.
